# Grand Rounds: Asbestos-Related Pericarditis in a Boiler Operator

**DOI:** 10.1289/ehp.10354

**Published:** 2007-09-11

**Authors:** Belayneh A. Abejie, Eugene H. Chung, Richard W. Nesto, Stefanos N. Kales

**Affiliations:** 1 Department of Environmental Health (Environmental & Occupational Medicine and Epidemiology), Harvard School of Public Health, Boston, Massachusetts, USA; 2 Department of Cardiovascular Medicine, Lahey Clinic, Burlington, Massachusetts, USA; 3 Department of Medicine, Harvard Medical School, Boston, Massachusetts, USA; 4 Employee Health & Industrial Medicine, Cambridge Health Alliance, Cambridge, Massachusetts, USA

**Keywords:** asbestos, boiler operators, calcific pericarditis, constrictive pericarditis, extrapulmonary

## Abstract

**Context:**

Occupational and environmental exposures to asbestos remain a public health problem even in developed countries. Because of the long latency in asbestos-related pathology, past asbestos exposure continues to contribute to incident disease. Asbestos most commonly produces pulmonary pathology, with asbestos-related pleural disease as the most common manifestation. Although the pleurae and pericardium share certain histologic characteristics, asbestos-related pericarditis is rarely reported.

**Case presentation:**

We present a 59-year-old man who worked around boilers for almost 30 years and was eventually determined to have calcific, constrictive pericarditis. He initially presented with an infectious exacerbation of chronic bronchitis. Chest radiographs demonstrated pleural and pericardial calcifications. Further evaluation with cardiac catheterization showed a hemodynamic picture consistent with constrictive pericarditis. A high-resolution computerized tomography scan of the chest demonstrated dense calcification in the pericardium, right pleural thickening and nodularity, right pleural plaque without calcification, and density in the right middle lobe. Pulmonary function testing showed mild obstruction and borderline low diffusing capacity.

**Discussion:**

Based on the patient’s occupational history, the presence of pleural pathology consistent with asbestos, previous evidence that asbestos can affect the pericardium, and absence of other likely explanations, we concluded that his pericarditis was asbestos-related.

**Relevance to clinical practice:**

Similar to pleural thickening and plaque formation, asbestos may cause progressive fibrosis of the pericardium.

A 59-year-old boiler worker saw his primary care physician for nasal congestion, dyspnea, and wheeze in mid-March 2005. Although he quit smoking 23 years earlier, he had a 20 pack-year history of smoking. Past medical history was significant for pneumonia (in 1991) and chronic bronchitis. The initial chest radiograph did not show pneumonia; however, it revealed noncircumferential calcification of the pericardium. The patient was treated for an infectious exacerbation of his chronic bronchitis with antibiotics and oral prednisone. After treatment for this episode, he attempted to return to work, but was unable to continue due to complaints of fatigue, shortness of breath, and exertional dyspnea. In April 2005, he consulted a pulmonologist who prescribed a combination inhaler (fluticasone and salmeterol).

Despite his pulmonary treatment, the patient continued to have fatigue, dyspnea, and occasional presyncopal events. As a result, he was referred to a cardiologist in May 2005. Physical examination was remarkable for obesity and distant heart sounds. Increased jugular venous pressure, Kussmaul’s sign, cyanosis, and peripheral edema were not present. His electrocardiogram showed sinus rhythm with occasional premature atrial beats. Review of chest radiographs revealed calcifications in the pleura and noncircumferential calcification of the pericardium. Echocardiogram showed mildly depressed left ventricular function and intermittent interventricular septal displacement in diastole, but no other findings diagnostic of pericardial constriction were noted. Based on a presumptive diagnosis of pericardial constriction, the patient underwent cardiac catheterization. Hemodynamic findings included intermittent equalization of right and left ventricular end diastolic filling pressures consistent with constrictive pericarditis (Figure 1). Partial calcification of the pericardium was seen under fluoroscopy. No obstructive coronary artery disease was present. The patient was treated with diuretics. He has remained stable, although with a limited capacity for any exertion.

The findings of pleural and pericardial calcification and the patient’s occupation raised questions with regard to asbestos as a possible etiology. The patient had worked around boilers for a power company from 1976 until leaving this job in March 2005. Starting as a utility man, he performed a variety of tasks with progressively increasing responsibilities in the boiler area, eventually becoming a boiler operator in 1992. During his tenure, he checked the pressures and temperature of water pipes, many of which were covered with asbestos insulation that was sometimes quite friable. Additionally, in the past he changed damaged packing valves, which were made of asbestos-containing material. Along with the asbestos, he was also exposed to fuel ash, sulfur dioxide, and other emissions. He stated that in the early years of his work respiratory protection was not provided. Records of workplace medical surveillance from the 1970s and 1980s were not available; however, in the 1990s the patient was restricted from wearing respirators and from fume (emissions) exposures because of his history of chronic bronchitis up until his final years with the company. Therefore, it is likely that during the majority of his career, he did not use respiratory protection. Although workplace spirometry had been performed on several occasions, the available records did not indicate any chest radiographs for pneumoconiosis surveillance. His previous occupational history included several other jobs but no known asbestos exposure. Environmental history failed to reveal additional asbestos exposures.

Further pulmonary evaluation included a high-resolution computerized tomography scan of the chest demonstrating dense calcification of the anterior pericardium (Figure 2); right pleural thickening and nodularity; right pleural plaque without calcification; right middle-lobe linear density consistent with scarring or atelectasis; and bilateral nodules, all > 5 mm in diameter. Pulmonary function testing in December 2006 demonstrated several abnormalities (Table 1). Total lung capacity (TLC) was normal, in spite of a markedly elevated residual volume (RV). The forced expiratory volume in 1 sec (FEV_1_) and FEV_1_/FVC (forced expiratory vital capacity) supported mild obstruction, and the increased RV and the RV/TLC ratio documented significant air trapping. The patient’s diffusing capacity was borderline and considered mildly decreased for that laboratory. There were moderate increases in the FVC, FEV_1_, and forced expiratory flow 25–75% after the administration of a bronchodilator, which suggested some reversibility, although these increases were not considered significant by standard criteria [American Thoracic Society (ATS) 1995; Celli and MacNee 2004]. A comparison with previous workplace spirometry between 1990 and 2004 did not show accelerated lung function loss but did demonstrate similar patterns with a previous FEV_1_ as low as 66% predicted.

In December 2006, as part of his workers compensation claim, the patient was referred to an occupational and environmental medicine physician by the Massachusetts Department of Industrial Accidents for an impartial examination. The purpose was to provide the responsible judge with independent opinions regarding diagnosis and causal relation to work. Based on the evidence presented above, the following diagnoses were reached: asbestos-related constrictive pericarditis, asbestos-related pleural disease, and mild chronic obstructive lung disease. Based on a reasonable degree of medical certainty, the impartial examiner concluded that these diagnoses were related to the patient’s asbestos exposures as a boiler worker.

## Discussion

The differential diagnosis of pericardial fibrosis is similar to that of pleural fibrosis and includes asbestos exposure, past tuberculosis, other infections, sarcoidosis, connective tissue disease, and trauma. None of these were apparent in our patient except asbestos exposure. Asbestos most commonly produces pulmonary pathology; however, asbestos bodies have been recovered from numerous extra-pulmonary tissues, including the heart (Auerbach et al. 1980; Churg et al. 1978). Asbestos pericarditis, albeit rarely reported, is a recognized extrapulmonary complication of past asbestos exposure (Davies et al. 1991; Fischbein et al. 1988; Jessurun et al. 1996; Trosini-Desert et al. 2003; Weg 1998; Weir and Gerstenhaber 2001). Other extra-pulmonary diseases associated with asbestos exposure include malignant mesothelioma of the peritoneum and pericardium, cancers of the colon, and retroperitoneal fibrosis (Beck et al. 1982; Browne and Smither 1983; Frumkin and Berlin 1988; Masse et al. 1980; Sebbag et al. 2000; Selikoff et al. 1964, 1979; Uibu et al. 2004). Proposed mechanisms of asbestos fiber migration into extrapulmonary tissues include direct spread and hematogenous and lymphatic transport (Auerbach et al. 1980). Given that the pleurae and pericardium share certain histologic characteristics and that asbestos-related pleural disease is the most common manifestation of past asbestos exposure, it is not surprising that progressive fibrosis of the pericardium, with or without calcification, may also result.

The hemodynamic picture in this patient is a consequence of diastolic restraint of right and left ventricular expansion imposed by an inelastic pericardium. The result is limited diastolic filling of both ventricles, as well as approximation of right and left filling patterns and end diastolic pressures, respectively. Echocardiogram also showed intermittent displacement of the interventricular septum in diastole. The classic “dip and plateau” diastolic filling pattern typically seen in pericardial constriction was not found in this case because the patient’s pericardial calcification was noncircumferential. The clinical correlation to past asbestos exposure was based on the occupational history, sufficient latency period, and other pathologic findings consistent with asbestos exposure.

Our patient was exposed to asbestos for almost 30 years while working in a power plant around boilers. Asbestos has long been used for thermal insulation in steam pipes and turbines. Fontaine and Trayer (1975) reported an area sample concentration of 4.7 fibers/cc at a Tennessee Valley Association power plant 30 years after an asbestos control effort started in 1945. Cammarano et al. (1984) found excess mortality among thermoelectric power plant workers where asbestos was used for insulation on the turbine boilers and pipes. Similarly, Breslow et al. (1954) observed 10 lung cancer cases in steam fitters, boilermakers, and asbestos workers, compared with 1 case in controls. Owen (1965) reported mesothelioma cases in laggers, pipe fitters, and boilermakers. In the Australian mesothelioma registry, between 1980 and 1985, boilermakers made up about 11% of the identified mesothelioma cases (Yeung and Rogers 2001).

Other findings in our patient that are compatible with asbestos exposure included pleural thickening and plaque; linear density in the lung parenchyma; and impaired lung function. Pleural plaques are a reliable bio-marker for establishing significant asbestos exposure (ATS 2004). Our patient also had chronic obstructive lung disease, which undoubtedly was significantly related to his history of past smoking and chronic bronchitis. Nonetheless, peribronchiolar fibrosis, which is associated with airflow obstruction, has been shown to be one of the earliest pathologic lesions in asbestos exposure (Begin et al. 1982; Bellis et al. 1989; Churg et al. 1985; Dumortier et al. 1990; Filipenko et al. 1985). Epidemiologic studies have also shown a higher prevalence of obstructive physiology (both decreased flows and air trapping) in asbestos-exposed workers than would be expected after adjustment for smoking (Becklake 1992; Griffith et al. 1993; Harless et al. 1978; Kilburn and Warshaw 1994; Rodriguez-Roisin et al. 1980). In addition, the finding of lung nodules, although small, warrant follow-up because the patient’s asbestos exposure puts him at increased risk of lung cancer (ATS 2004; Meurman et al. 1994; Oksa et al. 1997; Terra Filho et al. 2006; Ulvestad et al. 2002).

The pathogenesis of pericardial fibrosis is assumed to be similar to pleural or lung parenchyma fibrosis. Asbestos is persistent in the tissues. Settlement of the asbestos fibers is followed by influx of neutrophils and macrophages that generate hydrolytic enzymes, chemotaxins, cytokines, growth factors, and free radicals. Fibroblasts are activated as well. This cascade of events produces fibrosis, which increases as the fiber burden increases (ATS 2004; Schoenberger et al. 1982).

Although asbestos-related pericarditis due to occupational exposure is uncommon, a number of cases have been reported over the last 20 years (Table 2). Among the 12 cases listed in Table 2 (including our patient), all were > 40 years of age and most were > 60 years of age. Constrictive physiology was observed in 58% of the cases, with the remaining cases having pericardial effusions. All cases had associated pleural findings—mostly benign disease—but one patient had a mesothelioma. Most of the cases (83%) did not report definitive evidence of parenchymal asbestosis.

In addition, we are aware of at least two other reports of calcific pericarditis that were not related to occupational exposure but that do support asbestos as an etiology for pericardial fibrosis (Fraker et al. 1984; Weg 1998). These patients underwent a Beck procedure (a cardiac procedure used in the 1950s to develop collateral blood flow to the heart), which involved the introduction of asbestos into the pericardium to induce adhesion. In both cases, there was subsequent development of constrictive pericaridits with calcification (Fraker et al. 1984; Weg 1998).

Treatment for asbestos pericarditis is not different from that for other forms of constrictive pericarditis. This includes diuretics, salt restriction, and activity modification depending on the status of the patient. The definitive treatment is surgical decortication of the pericardium. We can only speculate whether workplace surveillance with chest radiography may have detected pleural changes and/or pericardial changes and, thus, led to removal from further exposure at an earlier stage. Because asbestos is persistent in the tissues, disease progression occurs even after the cessation of exposure. Therefore, we cannot say whether earlier removal from exposure would have produced a better outcome. Additionally, our patient’s condition, although stable at present, could worsen with additional fibrotic change of the pericardium and/or lung.

## Conclusion

The use and sale of asbestos have been restricted, and strict control measures are currently established in most industrialized countries; however, because of long latency and the chronic nature of asbestos-related diseases, we will continue to see patients with complications from past exposure. Therefore, careful workplace surveillance and attention to the occupational and environmental history in clinical practice are important for the early recognition of asbestos-related conditions. Although classically associated with pulmonary disease, asbestos fibers do reach other tissues and can produce extrapulmonary disease. Constrictive pericarditis is an uncommon complication of asbestos exposure. When found, it has been accompanied by asbestos-related pleural disease and occasionally other pathologic changes compatible with asbestos exposure.

## Figures and Tables

**Figure 1:**
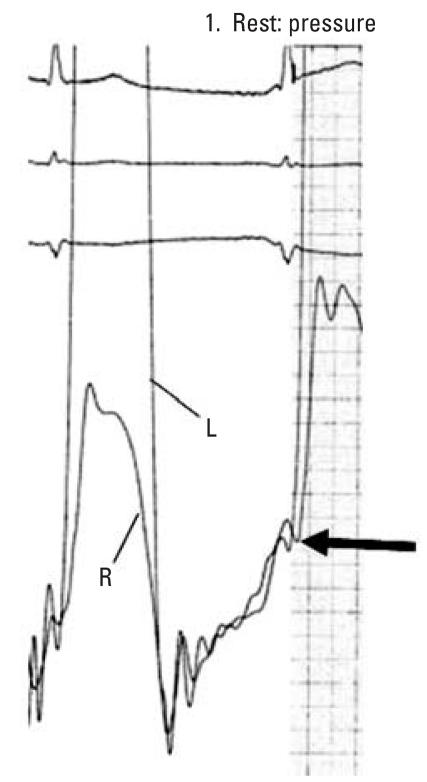
Hemodynamic tracing from cardiac catheterization. Simultaneous pressure measurements in the right (R) and left (L) ventricles demonstrate approximate equalization of diastolic filling pressures. End diastole is indicated by the arrow.

**Figure 2:**
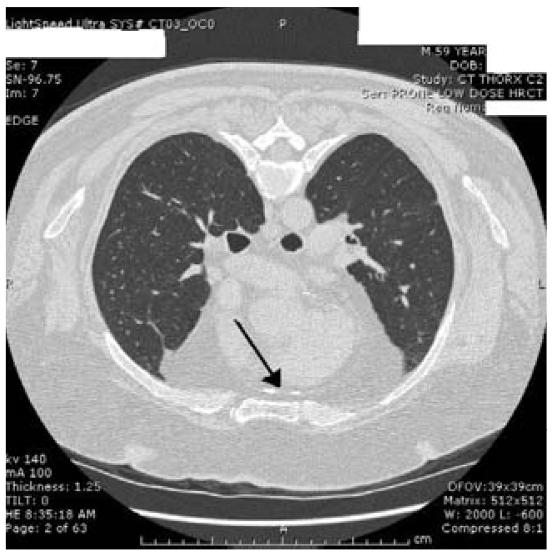
High-resolution computerized tomography scan image with patient in prone position demonstrating calcification of anterior pericardium.

**Table 1:** Lung function results, December 2006.

Pulmonary function test	Percent of predicted value	Percent change after bronchodilator
FEV_1_	70	7
FVC	79	9
FEV_1_/FVC%	71^a^	3
Forced expiratory flow 25–75%	49	29
TLC	98	
RV	140	
Carbon monoxide diffusing capacity (uncorrected)	76	

a Absolute value for FEV_1_/FVC%.

**Table 2:** Findings among published cases of pericarditis associated with occupational asbestos exposure.^a^

Reference	Patient’s age (years)	Occupation and total latency, years^b^	Pericardial findings	Pleural findings	Parenchymal lung findings
Fischbein et al. 1988	82	Boilermaker, 60 years	Calcification, constrictive physiology	Calcification	Interestitial fibrosis, asbestos bodies, and adenocarcinoma
Davies et al. 1991	43	Lagger (insulator), 25 years	Diffuse fibrous thickening, effusion	Calcified plaques, diffuse thickening	No asbestosis, but occasional asbestos bodies
Davies et al. 1991	64	Marine engine fitter, > 25 years	Thickening of the visceral and parietal layers, constrictive physiology	Calcified plaques, blood-stained effusion	Patchy interstitial fibrosis, classical crocidolite asbestos bodies
Davies et al. 1991	43	Asbestos mixer, 14 years	Diffuse dense fibrosis with fusion of visceral and parietal layers, constrictive physiology	Extensive pleural thickening, blood stained effusion	No asbestosis, only scanty asbestos bodies
Cordioli et al. 1994	73	Railroad worker, 40 years	Effusion and fibrohyaline plaques	Calcified plaques	None
Jessurun et al. 1996	56	Not reported	Effusion and diffuse thickening with inflammatory reaction	Calcified plaques	None
Trogrlic et al. 1997	60	Handling bags of asbestos at insulation company, 41 years	Effusion and diffuse inflammatory thickening	Bilateral benign asbestos effusions in past and subsequent diffuse thickening	Rounded atelectasis and bilateral blunting of the costophrenic angles
Weir and Gerstenhaber 2001	76	Retired telephone installer, unknown	Effusion, chronic inflammation and fibrosis, constrictive physiology	Mesethilioma, bilateral pleural effusion	Not reported
Trosini-Desert et al. 2003	62	Electric plant worker, 32 years	Pericardial thickening, constrictive physiology	Pleural thickening and diaphragmatic calcifications	Bilateral blunting of the costophrenic angles
Trosini-Desert et al. 2003	76	Lagger (insulator), 20 years	Cirucmferential pericardial thickening with some calcification, constrictive physiology	Calcified pleural plaques and thickening	Atelectasis
Roggeri et al. 2003	60	Not specified	Hemorrhagic effusion	Bilateral plaques	None
Present study	59	Boiler operator, 39 years	Pericardial calcification, constrictive physiology	Plaques and thickening	Right middle lobe scarring, bilateral small nodules

a Adapted in part from Trosini-Desert et al. (2003).

b Approximate time from first exposure to time of presentation.
